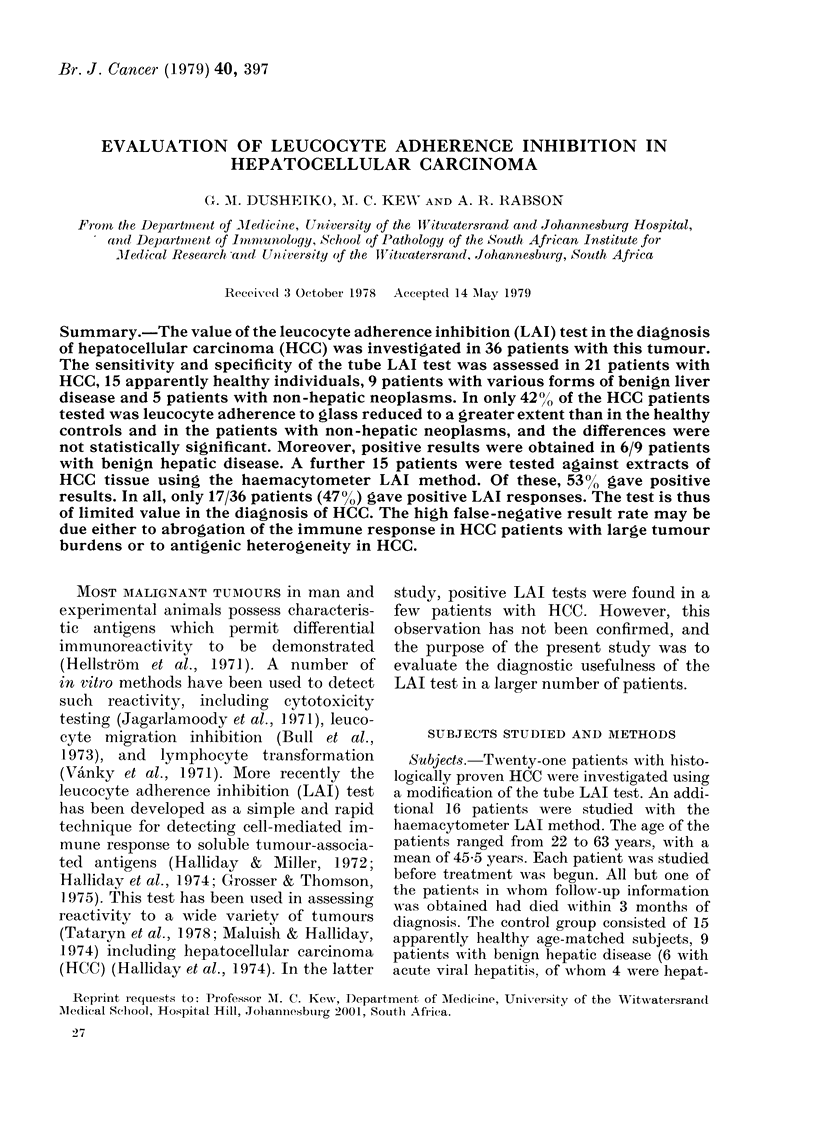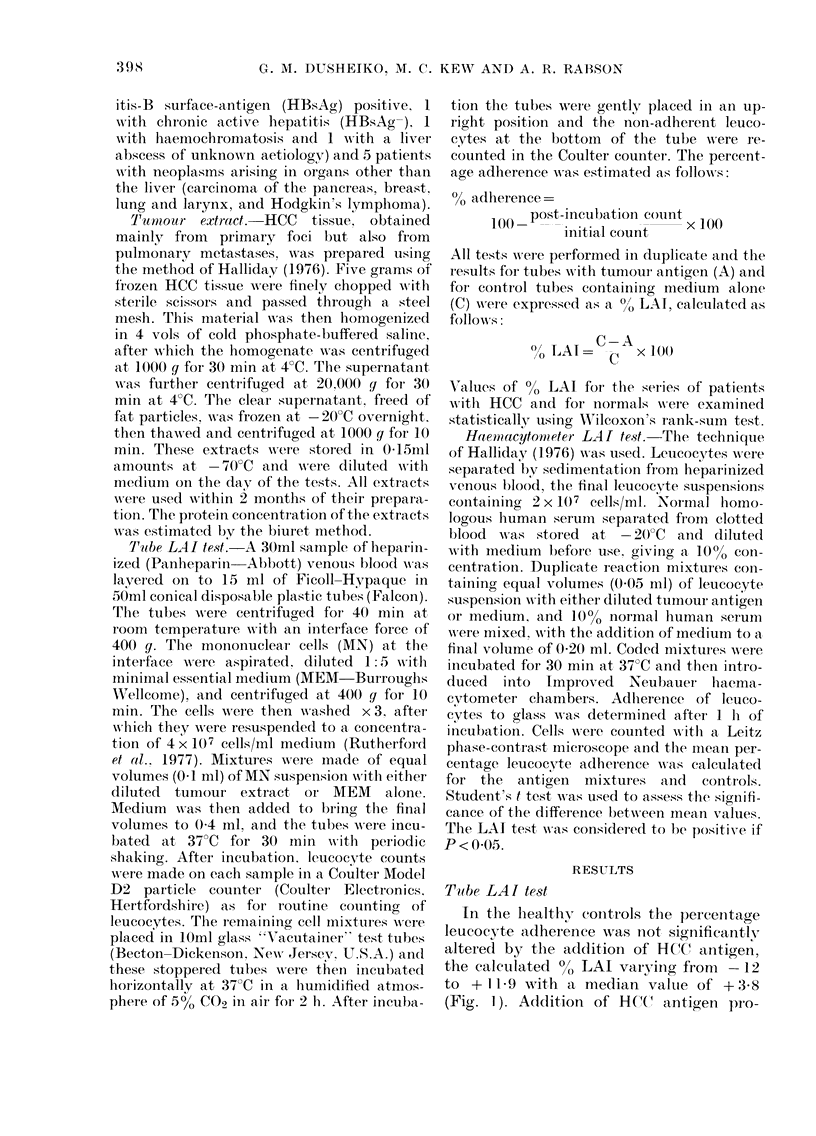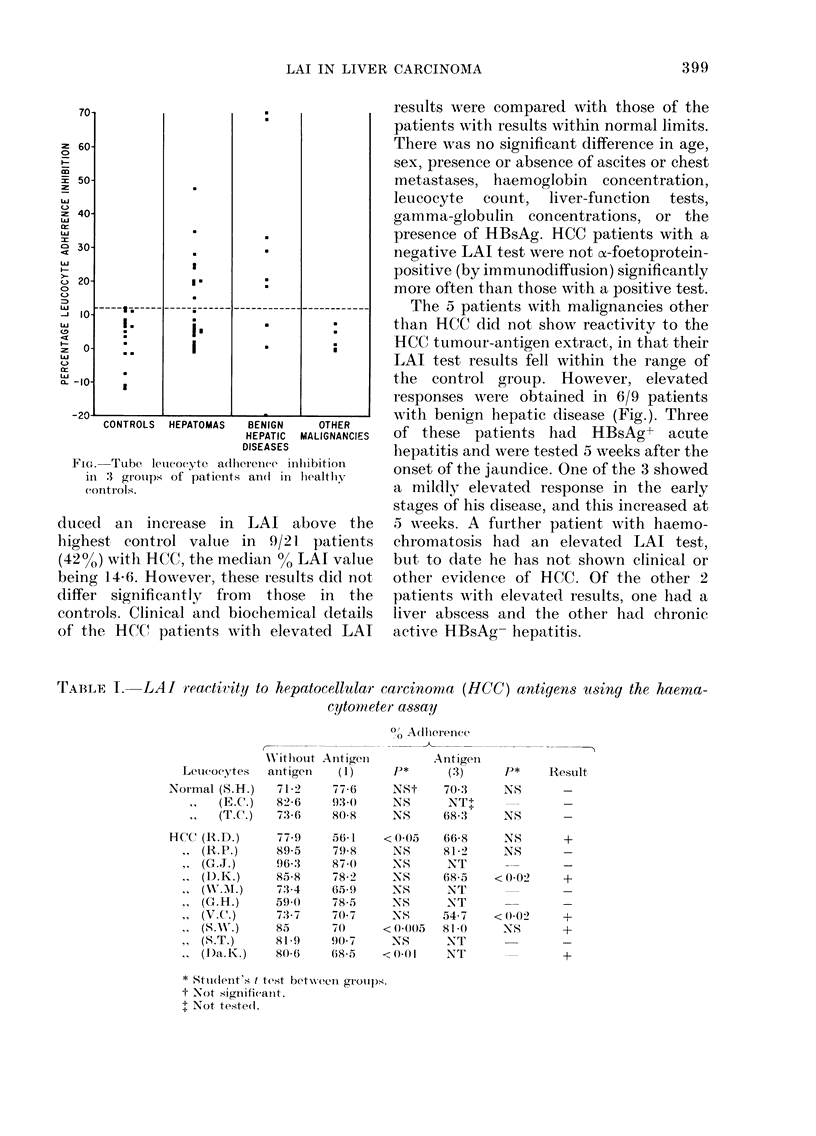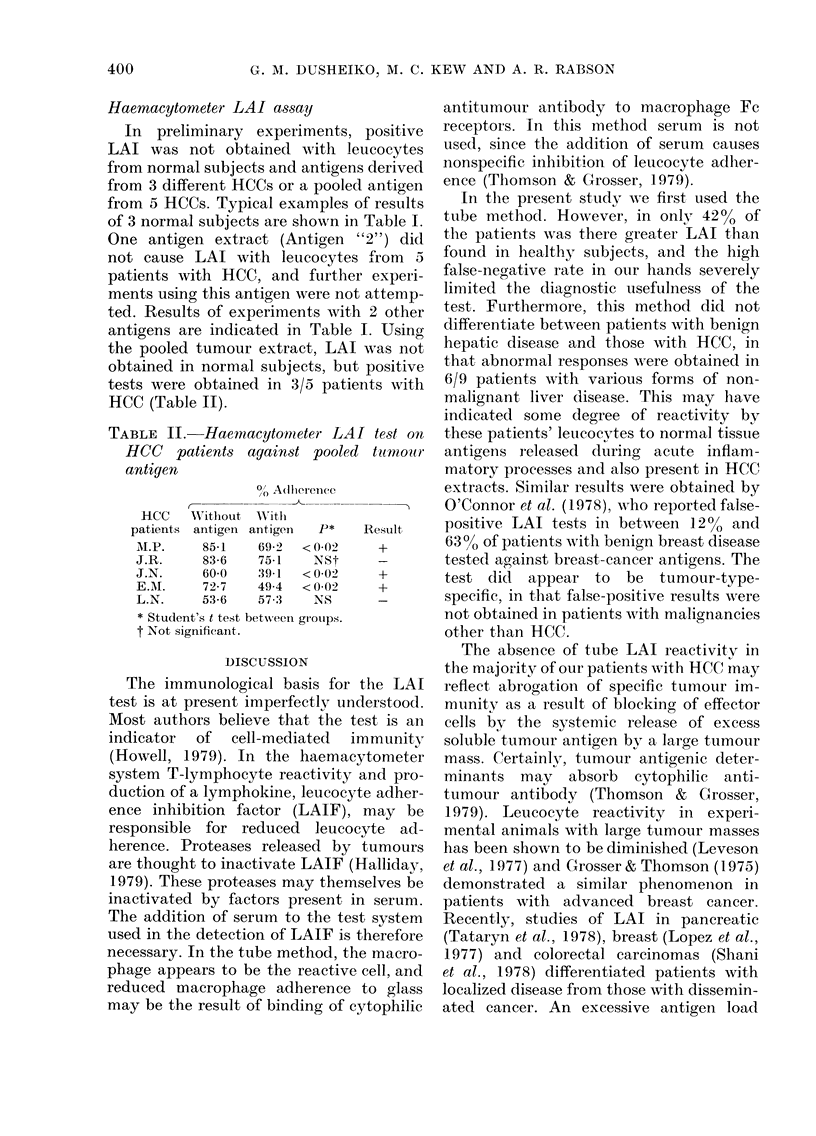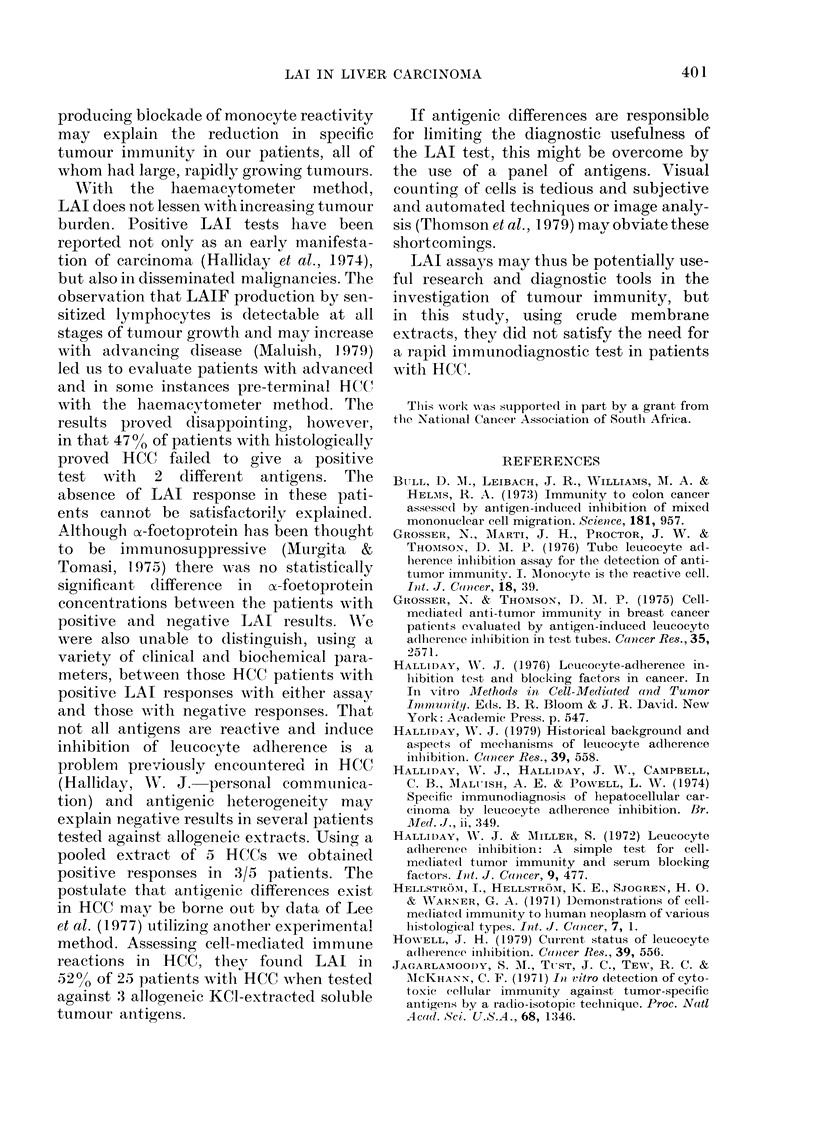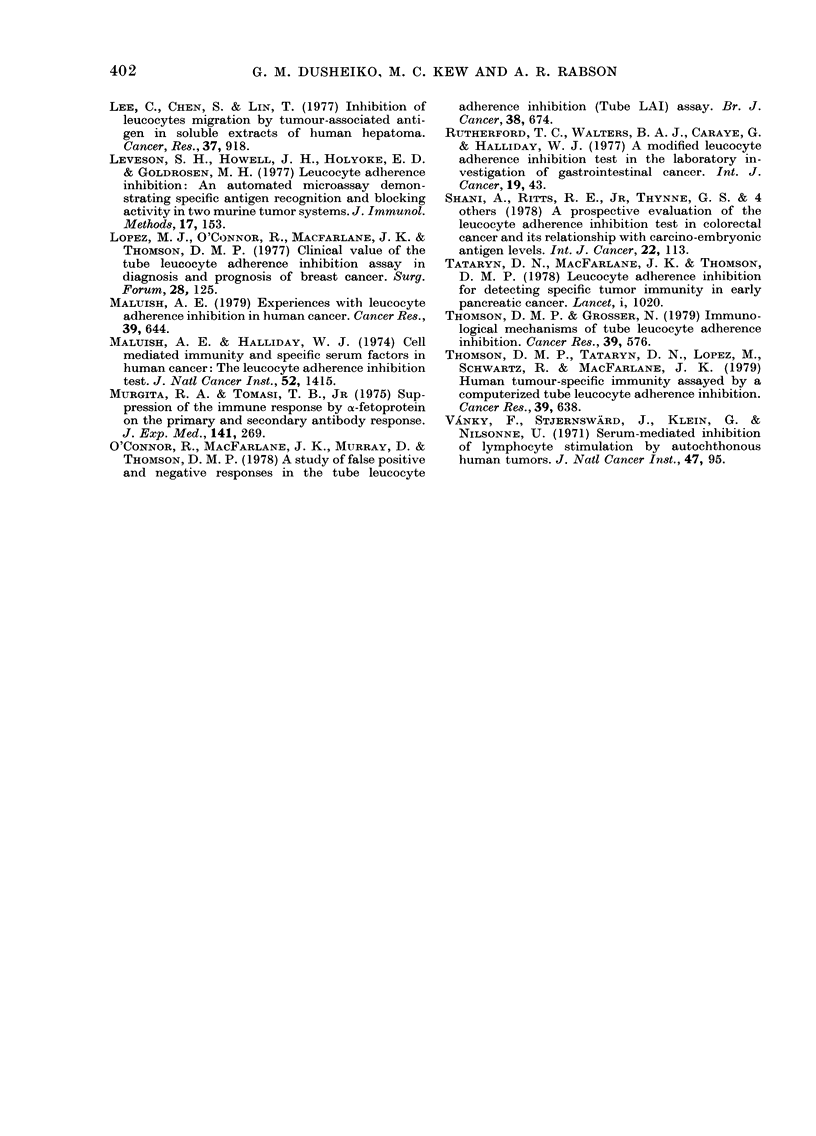# Evaluation of leucocyte adherence inhibition in hepatocellular carcinoma.

**DOI:** 10.1038/bjc.1979.194

**Published:** 1979-09

**Authors:** G. M. Dusheiko, M. C. Kew, A. R. Rabson

## Abstract

The value of the leucocyte adherence inhibition (LAI) test in the diagnosis of hepatocellular carcinoma (HCG) was investigated in 36 patients with this tumour. The sensitivity and specificity of the tube LAI test was assessed in 21 patients with HCC, 15 apparently healthy individuals, 9 patients with various forms of benign liver disease and 5 patients with non-hepatic neoplasms. In only 42% of the HCC patients tested was leucocyte adherence to glass reduced to a greater extent than in the healthy controls and in the patients with non-hepatic neoplasms, and the differences were not statistically significant. Moreover, positive results were obtained in 6/9 patients with benign hepatic disease. A further 15 patients were tested against extracts of HCC tissue using the haemacytometer LAI method. Of these, 53% gave positive results. In all, only 17/36 patients (47%) gave positive LAI responses. The test is thus of limited value in the diagnosis of HCC. The high false-negative result rate may be due either to abrogation of the immune response in HCC patients with large tumour burdens or to antigenic heterogeneity in HCC.


*Br. J. Cancer* (1979) **40**, 397

### EVALUATION OF LEUCOCYTE ADHERENCE INHIBITION IN HEPATOCELLULAR CARCINOMA

G. M. DUSHEIKO, M. C. KEW AND A. R. RABSON

*From the Department of Medicine, University of the Witwatersrand and Johannesburg Hospital, and Department of Immunology, School of Pathology of the South African Institute for Medical Research and University of the Witwatersrand, Johannesburg, South Africa*

Received 3 October 1978 Accepted 14 May 1979

**Summary.—The value of the leucocyte adherence inhibition (LAI) test in the diagnosis of hepatocellular carcinoma (HCC) was investigated in 36 patients with this tumour. The sensitivity and specificity of the tube LAI test was assessed in 21 patients with HCC, 15 apparently healthy individuals, 9 patients with various forms of benign liver disease and 5 patients with non-hepatic neoplasms. In only 42% of the HCC patients tested was leucocyte adherence to glass reduced to a greater extent than in the healthy controls and in the patients with non-hepatic neoplasms, and the differences were not statistically significant. Moreover, positive results were obtained in 6/9 patients with benign hepatic disease. A further 15 patients were tested against extracts of HCC tissue using the haemacytometer LAI method. Of these, 53% gave positive results. In all, only 17/36 patients (47%) gave positive LAI responses. The test is thus of limited value in the diagnosis of HCC. The high false-negative result rate may be due either to abrogation of the immune response in HCC patients with large tumour burdens or to antigenic heterogeneity in HCC.**

MOST MALIGNANT TUMOURS in man and experimental animals possess characteristic antigens which permit differential immunoreactivity to be demonstrated (Hellström *et al.*, 1971). A number of *in vitro* methods have been used to detect such reactivity, including cytotoxicity testing (Jagarlamoody *et al.*, 1971), leucocyte migration inhibition (Bull *et al.*, 1973), and lymphocyte transformation (Vánky *et al.*, 1971). More recently the leucocyte adherence inhibition (LAI) test has been developed as a simple and rapid technique for detecting cell-mediated immune response to soluble tumour-associated antigens (Halliday & Miller, 1972; Halliday *et al.*, 1974; Grosser & Thomson, 1975). This test has been used in assessing reactivity to a wide variety of tumours (Tataryn *et al.*, 1978; Maluish & Halliday, 1974) including hepatocellular carcinoma (HCC) (Halliday *et al.*, 1974). In the latter study, positive LAI tests were found in a few patients with HCC. However, this observation has not been confirmed, and the purpose of the present study was to evaluate the diagnostic usefulness of the LAI test in a larger number of patients.

### SUBJECTS STUDIED AND METHODS

*Subjects.*—Twenty-one patients with histologically proven HCC were investigated using a modification of the tube LAI test. An additional 16 patients were studied with the haemacytometer LAI method. The age of the patients ranged from 22 to 63 years, with a mean of 45·5 years. Each patient was studied before treatment was begun. All but one of the patients in whom follow-up information was obtained had died within 3 months of diagnosis. The control group consisted of 15 apparently healthy age-matched subjects, 9 patients with benign hepatic disease (6 with acute viral hepatitis, of whom 4 were hepat-

Reprint requests to: Professor M. C. Kew, Department of Medicine, University of the Witwatersrand Medical School, Hospital Hill, Johannesburg 2001, South Africa.

itis-B surface-antigen (HBsAg) positive, 1 with chronic active hepatitis (HBsAg$^-$), 1 with haemochromatosis and 1 with a liver abscess of unknown aetiology) and 5 patients with neoplasms arising in organs other than the liver (carcinoma of the pancreas, breast, lung and larynx, and Hodgkin's lymphoma).

*Tumour extract.*—HCC tissue, obtained mainly from primary foci but also from pulmonary metastases, was prepared using the method of Halliday (1976). Five grams of frozen HCC tissue were finely chopped with sterile scissors and passed through a steel mesh. This material was then homogenized in 4 vols of cold phosphate-buffered saline, after which the homogenate was centrifuged at 1000 *g* for 30 min at 4°C. The supernatant was further centrifuged at 20,000 *g* for 30 min at 4°C. The clear supernatant, freed of fat particles, was frozen at −20°C overnight, then thawed and centrifuged at 1000 *g* for 10 min. These extracts were stored in 0·15ml amounts at −70°C and were diluted with medium on the day of the tests. All extracts were used within 2 months of their preparation. The protein concentration of the extracts was estimated by the biuret method.

*Tube LAI test.*—A 30ml sample of heparinized (Panheparin—Abbott) venous blood was layered on to 15 ml of Ficoll–Hypaque in 50ml conical disposable plastic tubes (Falcon). The tubes were centrifuged for 40 min at room temperature with an interface force of 400 *g*. The mononuclear cells (MN) at the interface were aspirated, diluted 1:5 with minimal essential medium (MEM—Burroughs Wellcome), and centrifuged at 400 *g* for 10 min. The cells were then washed ×3, after which they were resuspended to a concentration of $4\times10^7$ cells/ml medium (Rutherford *et al.*, 1977). Mixtures were made of equal volumes (0·1 ml) of MN suspension with either diluted tumour extract or MEM alone. Medium was then added to bring the final volumes to 0·4 ml, and the tubes were incubated at 37°C for 30 min with periodic shaking. After incubation, leucocyte counts were made on each sample in a Coulter Model D2 particle counter (Coulter Electronics, Hertfordshire) as for routine counting of leucocytes. The remaining cell mixtures were placed in 10ml glass "Vacutainer" test tubes (Becton–Dickenson, New Jersey, U.S.A.) and these stoppered tubes were then incubated horizontally at 37°C in a humidified atmosphere of 5% $CO_2$ in air for 2 h. After incubation the tubes were gently placed in an upright position and the non-adherent leucocytes at the bottom of the tube were recounted in the Coulter counter. The percentage adherence was estimated as follows:

$$\% \text{ adherence} = 100 - \frac{\text{post-incubation count}}{\text{initial count}} \times 100$$

All tests were performed in duplicate and the results for tubes with tumour antigen (A) and for control tubes containing medium alone (C) were expressed as a % LAI, calculated as follows:

$$\% \text{ LAI} = \frac{C-A}{C} \times 100$$

Values of % LAI for the series of patients with HCC and for normals were examined statistically using Wilcoxon's rank-sum test.

*Haemacytometer LAI test.*—The technique of Halliday (1976) was used. Leucocytes were separated by sedimentation from heparinized venous blood, the final leucocyte suspensions containing $2\times10^7$ cells/ml. Normal homologous human serum separated from clotted blood was stored at −20°C and diluted with medium before use, giving a 10% concentration. Duplicate reaction mixtures containing equal volumes (0·05 ml) of leucocyte suspension with either diluted tumour antigen or medium, and 10% normal human serum were mixed, with the addition of medium to a final volume of 0·20 ml. Coded mixtures were incubated for 30 min at 37°C and then introduced into Improved Neubauer haemacytometer chambers. Adherence of leucocytes to glass was determined after 1 h of incubation. Cells were counted with a Leitz phase-contrast microscope and the mean percentage leucocyte adherence was calculated for the antigen mixtures and controls. Student's *t* test was used to assess the significance of the difference between mean values. The LAI test was considered to be positive if $P<0{\cdot}05$.

### RESULTS

### *Tube LAI test*

In the healthy controls the percentage leucocyte adherence was not significantly altered by the addition of HCC antigen, the calculated % LAI varying from −12 to +11·9 with a median value of +3·8 (Fig. 1). Addition of HCC antigen pro-

FIG.—Tube leucocyte adherence inhibition in 3 groups of patients and in healthy controls.

duced an increase in LAI above the highest control value in 9/21 patients (42%) with HCC, the median % LAI value being 14·6. However, these results did not differ significantly from those in the controls. Clinical and biochemical details of the HCC patients with elevated LAI results were compared with those of the patients with results within normal limits. There was no significant difference in age, sex, presence or absence of ascites or chest metastases, haemoglobin concentration, leucocyte count, liver-function tests, gamma-globulin concentrations, or the presence of HBsAg. HCC patients with a negative LAI test were not α-foetoprotein-positive (by immunodiffusion) significantly more often than those with a positive test.

The 5 patients with malignancies other than HCC did not show reactivity to the HCC tumour-antigen extract, in that their LAI test results fell within the range of the control group. However, elevated responses were obtained in 6/9 patients with benign hepatic disease (Fig.). Three of these patients had HBsAg+ acute hepatitis and were tested 5 weeks after the onset of the jaundice. One of the 3 showed a mildly elevated response in the early stages of his disease, and this increased at 5 weeks. A further patient with haemochromatosis had an elevated LAI test, but to date he has not shown clinical or other evidence of HCC. Of the other 2 patients with elevated results, one had a liver abscess and the other had chronic active HBsAg− hepatitis.

TABLE I.—*LAI reactivity to hepatocellular carcinoma (HCC) antigens using the haemacytometer assay*

| | % Adherence | | | | | |
|---|---|---|---|---|---|---|
| Leucocytes | Without antigen | Antigen (1) | *P** | Antigen (3) | *P** | Result |
| Normal (S.H.) | 71·2 | 77·6 | NS† | 70·3 | NS | − |
| ,, (E.C.) | 82·6 | 93·0 | NS | NT‡ | — | − |
| ,, (T.C.) | 73·6 | 80·8 | NS | 68·3 | NS | − |
| HCC (R.D.) | 77·9 | 56·1 | < 0·05 | 66·8 | NS | + |
| ,, (R.P.) | 89·5 | 79·8 | NS | 81·2 | NS | − |
| ,, (G.J.) | 96·3 | 87·0 | NS | NT | — | − |
| ,, (D.K.) | 85·8 | 78·2 | NS | 68·5 | < 0·02 | + |
| ,, (W.M.) | 73·4 | 65·9 | NS | NT | — | − |
| ,, (G.H.) | 59·0 | 78·5 | NS | NT | — | − |
| ,, (V.C.) | 73·7 | 70·7 | NS | 54·7 | < 0·02 | + |
| ,, (S.W.) | 85 | 70 | < 0·005 | 81·0 | NS | + |
| ,, (S.T.) | 81·9 | 90·7 | NS | NT | — | − |
| ,, (Da.K.) | 80·6 | 68·5 | < 0·01 | NT | — | + |

* Student's *t* test between groups.

† Not significant.

‡ Not tested.

*Haemacytometer LAI assay*

In preliminary experiments, positive LAI was not obtained with leucocytes from normal subjects and antigens derived from 3 different HCCs or a pooled antigen from 5 HCCs. Typical examples of results of 3 normal subjects are shown in Table I. One antigen extract (Antigen "2") did not cause LAI with leucocytes from 5 patients with HCC, and further experiments using this antigen were not attempted. Results of experiments with 2 other antigens are indicated in Table I. Using the pooled tumour extract, LAI was not obtained in normal subjects, but positive tests were obtained in 3/5 patients with HCC (Table II).

TABLE II.—*Haemacytometer LAI test on HCC patients against pooled tumour antigen*

| | % Adherence | | | |
|---|---|---|---|---|
| HCC patients | Without antigen | With antigen | $P$* | Result |
| M.P. | 85·1 | 69·2 | <0·02 | + |
| J.R. | 83·6 | 75·1 | NS† | − |
| J.N. | 60·0 | 39·1 | <0·02 | + |
| E.M. | 72·7 | 49·4 | <0·02 | + |
| L.N. | 53·6 | 57·3 | NS | − |

* Student's $t$ test between groups.

† Not significant.

### DISCUSSION

The immunological basis for the LAI test is at present imperfectly understood. Most authors believe that the test is an indicator of cell-mediated immunity (Howell, 1979). In the haemacytometer system T-lymphocyte reactivity and production of a lymphokine, leucocyte adherence inhibition factor (LAIF), may be responsible for reduced leucocyte adherence. Proteases released by tumours are thought to inactivate LAIF (Halliday, 1979). These proteases may themselves be inactivated by factors present in serum. The addition of serum to the test system used in the detection of LAIF is therefore necessary. In the tube method, the macrophage appears to be the reactive cell, and reduced macrophage adherence to glass may be the result of binding of cytophilic antitumour antibody to macrophage Fc receptors. In this method serum is not used, since the addition of serum causes nonspecific inhibition of leucocyte adherence (Thomson & Grosser, 1979).

In the present study we first used the tube method. However, in only 42% of the patients was there greater LAI than found in healthy subjects, and the high false-negative rate in our hands severely limited the diagnostic usefulness of the test. Furthermore, this method did not differentiate between patients with benign hepatic disease and those with HCC, in that abnormal responses were obtained in 6/9 patients with various forms of nonmalignant liver disease. This may have indicated some degree of reactivity by these patients' leucocytes to normal tissue antigens released during acute inflammatory processes and also present in HCC extracts. Similar results were obtained by O'Connor *et al.* (1978), who reported false-positive LAI tests in between 12% and 63% of patients with benign breast disease tested against breast-cancer antigens. The test did appear to be tumour-type-specific, in that false-positive results were not obtained in patients with malignancies other than HCC.

The absence of tube LAI reactivity in the majority of our patients with HCC may reflect abrogation of specific tumour immunity as a result of blocking of effector cells by the systemic release of excess soluble tumour antigen by a large tumour mass. Certainly, tumour antigenic determinants may absorb cytophilic antitumour antibody (Thomson & Grosser, 1979). Leucocyte reactivity in experimental animals with large tumour masses has been shown to be diminished (Leveson *et al.*, 1977) and Grosser & Thomson (1975) demonstrated a similar phenomenon in patients with advanced breast cancer. Recently, studies of LAI in pancreatic (Tataryn *et al.*, 1978), breast (Lopez *et al.*, 1977) and colorectal carcinomas (Shani *et al.*, 1978) differentiated patients with localized disease from those with disseminated cancer. An excessive antigen load producing blockade of monocyte reactivity may explain the reduction in specific tumour immunity in our patients, all of whom had large, rapidly growing tumours.

With the haemacytometer method, LAI does not lessen with increasing tumour burden. Positive LAI tests have been reported not only as an early manifestation of carcinoma (Halliday *et al.*, 1974), but also in disseminated malignancies. The observation that LAIF production by sensitized lymphocytes is detectable at all stages of tumour growth and may increase with advancing disease (Maluish, 1979) led us to evaluate patients with advanced and in some instances pre-terminal HCC with the haemacytometer method. The results proved disappointing, however, in that 47% of patients with histologically proved HCC failed to give a positive test with 2 different antigens. The absence of LAI response in these patients cannot be satisfactorily explained. Although $\alpha$-foetoprotein has been thought to be immunosuppressive (Murgita & Tomasi, 1975) there was no statistically significant difference in $\alpha$-foetoprotein concentrations between the patients with positive and negative LAI results. We were also unable to distinguish, using a variety of clinical and biochemical parameters, between those HCC patients with positive LAI responses with either assay and those with negative responses. That not all antigens are reactive and induce inhibition of leucocyte adherence is a problem previously encountered in HCC (Halliday, W. J.—personal communication) and antigenic heterogeneity may explain negative results in several patients tested against allogeneic extracts. Using a pooled extract of 5 HCCs we obtained positive responses in 3/5 patients. The postulate that antigenic differences exist in HCC may be borne out by data of Lee *et al.* (1977) utilizing another experimental method. Assessing cell-mediated immune reactions in HCC, they found LAI in 52% of 25 patients with HCC when tested against 3 allogeneic KCl-extracted soluble tumour antigens.

If antigenic differences are responsible for limiting the diagnostic usefulness of the LAI test, this might be overcome by the use of a panel of antigens. Visual counting of cells is tedious and subjective and automated techniques or image analysis (Thomson *et al.*, 1979) may obviate these shortcomings.

LAI assays may thus be potentially useful research and diagnostic tools in the investigation of tumour immunity, but in this study, using crude membrane extracts, they did not satisfy the need for a rapid immunodiagnostic test in patients with HCC.

This work was supported in part by a grant from the National Cancer Association of South Africa.

### REFERENCES

BULL, D. M., LEIBACH, J. R., WILLIAMS, M. A. & HELMS, R. A. (1973) Immunity to colon cancer assessed by antigen-induced inhibition of mixed mononuclear cell migration. *Science*, **181**, 957.

GROSSER, N., MARTI, J. H., PROCTOR, J. W. & THOMSON, D. M. P. (1976) Tube leucocyte adherence inhibition assay for the detection of anti-tumor immunity. I. Monocyte is the reactive cell. *Int. J. Cancer*, **18**, 39.

GROSSER, N. & THOMSON, D. M. P. (1975) Cell-mediated anti-tumor immunity in breast cancer patients evaluated by antigen-induced leucocyte adherence inhibition in test tubes. *Cancer Res.*, **35**, 2571.

HALLIDAY, W. J. (1976) Leucocyte-adherence inhibition test and blocking factors in cancer. In In vitro *Methods in Cell-Mediated and Tumor Immunity*. Eds. B. R. Bloom & J. R. David. New York: Academic Press. p. 547.

HALLIDAY, W. J. (1979) Historical background and aspects of mechanisms of leucocyte adherence inhibition. *Cancer Res.*, **39**, 558.

HALLIDAY, W. J., HALLIDAY, J. W., CAMPBELL, C. B., MALUISH, A. E. & POWELL, L. W. (1974) Specific immunodiagnosis of hepatocellular carcinoma by leucocyte adherence inhibition. *Br. Med. J.*, ii, 349.

HALLIDAY, W. J. & MILLER, S. (1972) Leucocyte adherence inhibition: A simple test for cell-mediated tumor immunity and serum blocking factors. *Int. J. Cancer*, **9**, 477.

HELLSTRÖM, I., HELLSTRÖM, K. E., SJOGREN, H. O. & WARNER, G. A. (1971) Demonstrations of cell-mediated immunity to human neoplasm of various histological types. *Int. J. Cancer*, **7**, 1.

HOWELL, J. H. (1979) Current status of leucocyte adherence inhibition. *Cancer Res.*, **39**, 556.

JAGARLAMOODY, S. M., TUST, J. C., TEW, R. C. & MCKHANN, C. F. (1971) *In vitro* detection of cytotoxic cellular immunity against tumor-specific antigens by a radio-isotopic technique. *Proc. Natl Acad. Sci. U.S.A.*, **68**, 1346.

LEE, C., CHEN, S. & LIN, T. (1977) Inhibition of leucocytes migration by tumour-associated antigen in soluble extracts of human hepatoma. *Cancer, Res.*, **37**, 918.

LEVESON, S. H., HOWELL, J. H., HOLYOKE, E. D. & GOLDROSEN, M. H. (1977) Leucocyte adherence inhibition: An automated microassay demonstrating specific antigen recognition and blocking activity in two murine tumor systems. *J. Immunol. Methods*, **17**, 153.

LOPEZ, M. J., O'CONNOR, R., MACFARLANE, J. K. & THOMSON, D. M. P. (1977) Clinical value of the tube leucocyte adherence inhibition assay in diagnosis and prognosis of breast cancer. *Surg. Forum*, **28**, 125.

MALUISH, A. E. (1979) Experiences with leucocyte adherence inhibition in human cancer. *Cancer Res.*, **39**, 644.

MALUISH, A. E. & HALLIDAY, W. J. (1974) Cell mediated immunity and specific serum factors in human cancer: The leucocyte adherence inhibition test. *J. Natl Cancer Inst.*, **52**, 1415.

MURGITA, R. A. & TOMASI, T. B., JR (1975) Suppression of the immune response by α-fetoprotein on the primary and secondary antibody response. *J. Exp. Med.*, **141**, 269.

O'CONNOR, R., MACFARLANE, J. K., MURRAY, D. & THOMSON, D. M. P. (1978) A study of false positive and negative responses in the tube leucocyte adherence inhibition (Tube LAI) assay. *Br. J. Cancer*, **38**, 674.

RUTHERFORD, T. C., WALTERS, B. A. J., CARAYE, G. & HALLIDAY, W. J. (1977) A modified leucocyte adherence inhibition test in the laboratory investigation of gastrointestinal cancer. *Int. J. Cancer*, **19**, 43.

SHANI, A., RITTS, R. E., JR, THYNNE, G. S. & 4 others (1978) A prospective evaluation of the leucocyte adherence inhibition test in colorectal cancer and its relationship with carcino-embryonic antigen levels. *Int. J. Cancer*, **22**, 113.

TATARYN, D. N., MACFARLANE, J. K. & THOMSON, D. M. P. (1978) Leucocyte adherence inhibition for detecting specific tumor immunity in early pancreatic cancer. *Lancet*, i, 1020.

THOMSON, D. M. P. & GROSSER, N. (1979) Immunological mechanisms of tube leucocyte adherence inhibition. *Cancer Res.*, **39**, 576.

THOMSON, D. M. P., TATARYN, D. N., LOPEZ, M., SCHWARTZ, R. & MACFARLANE, J. K. (1979) Human tumour-specific immunity assayed by a computerized tube leucocyte adherence inhibition. *Cancer Res.*, **39**, 638.

VÁNKY, F., STJERNSWÄRD, J., KLEIN, G. & NILSONNE, U. (1971) Serum-mediated inhibition of lymphocyte stimulation by autochthonous human tumors. *J. Natl Cancer Inst.*, **47**, 95.